# Combination of Intravitreal Injection of Ranibizumab and Photocoagulation for the Treatment of Aggressive Posterior Retinopathy of Prematurity with Vitreous Hemorrhage

**DOI:** 10.1155/2016/5029278

**Published:** 2016-12-14

**Authors:** Yu Xu, Xiaoli Kang, Qi Zhang, Qiujing Huang, Jiao Lv, Peiquan Zhao

**Affiliations:** Department of Ophthalmology, Xinhua Hospital affiliated to Shanghai Jiao Tong University School of Medicine, Shanghai 200092, China

## Abstract

To investigate the efficacy of intravitreal ranibizumab (IVR) combined with laser photocoagulation for aggressive posterior retinopathy of prematurity (AP-ROP) patients with vitreous hemorrhage, we conducted a retrospective observational case series study. A total of 37 eyes of 20 patients' medical records were reviewed. Patients first received IVR (0.25 mg/0.025 mL) and later photocoagulation. The mean postconceptual age of injection was 34.6 ± 1.4 weeks, and the mean follow-up period was 39.3 ± 8.3 weeks. During the follow-up, 96.6% eyes had various degree of rapid absorption of vitreous hemorrhage after IVR. The mean time of received first photocoagulation after IVR was 4.8 ± 2.9 weeks. Ten (27.0%) eyes received second laser therapy and the mean time of second laser therapy after IVR was 3.2 ± 0.8 weeks. All eyes exhibited adequate regression of ROP and were stable with attached retina. Fibrosis membrane was observed in seven eyes (18.9%) and three of them demonstrated mild ectopic macula. No significant side effects related to IVR were observed. So IVR could be conducted as primary treatment of AP-ROP associated with vitreous hemorrhage, which can improve the fundus visibility, followed by conventional photocoagulation. Further randomized controlled trials are necessary to compare the clinical efficacy and safety with conventional interventions.

## 1. Introduction

Retinopathy of prematurity (ROP), which is a major cause of visual impairment in children, is a vasoproliferative disorder associated with premature birth [1]. Laser photocoagulation is the gold standard treatment for proliferative ROP and has proven useful in reducing progression of classic ROP [2, 3]. However, in treating aggressive posterior retinopathy of prematurity (AP-ROP), as a more severe and unusual form of ROP, laser photocoagulation often fails to stop its progression to retinal detachment even with timely and complete treatment [4, 5]. Compared with classic ROP, AP-ROP is more likely associated with vitreous hemorrhage. The presence of vitreous hemorrhage often makes the completion of laser treatment more difficult due to the poor fundus visibility and is always associated with higher rates of unfavorable outcomes [4, 6, 7]. So how to treat these patients in a more efficacious way poses a real challenge to pediatric ophthalmologists.

Previous studies demonstrated that the vascular endothelial growth factor (VEGF) is a key factor in the progression of ROP [3]. Directly halting the VEGF molecules released from the ischemic retina, intravitreal injection of anti-VEGF agents, either with bevacizumab (Avastin®; Genentech Inc.) or Ranibizumab (Lucentis®; Novartis), was demonstrated as effective in treating severe ROP and thus gained increasing popularity [8–11]. Main advantages of anti-VEGF treatment over conventional laser photocoagulation include causing rapid regression of acute-phase ROP (neovascularization and plus disease), allowing potentials for retinal vascularization, approaching eyes with a rigid pupil, and reducing the risks of unfavorable outcomes in zone I or posterior zone II ROP [8, 9, 11].

Our purpose of this study was to investigate the efficacy of intravitreal injection of ranibizumab (IVR) combined with laser photocoagulation for the treatment of aggressive posterior retinopathy of prematurity (AP-ROP) patients with vitreous hemorrhage.

## 2. Methods

The design and execution of this retrospective noncomparative observational study was approved by Xinhua Hospital affiliated to Shanghai Jiao Tong University School of Medicine Institutional Review Board. The study protocol adhered to the tenets of the Declaration of Helsinki. Written informed consent was obtained from all participants' parents or guardians.

### 2.1. Patients

Thirty-seven eyes of twenty patients having a primary diagnosis of AP-ROP with vitreous hemorrhage obscuring the posterior pole or obscuring at least 4 contiguous clock hours of disease at the junction of vascular and avascular retina at Xinhua Hospital from April 2013 to March 2015 were enrolled. The medical records were carefully reviewed. AP-ROP patients without primary vitreous hemorrhage or with vitreous hemorrhage do not meet the above criteria, or the patients with incomplete contents of chart were excluded.

### 2.2. Diagnosis and Classification of ROP

The diagnosis of AP-ROP was according to the international classification of retinopathy of prematurity (ICROP, 2005) [12]. AP-ROP was defined as a flat network of neovascularization in posterior pole associated with increased dilation and tortuosity in all 4 quadrants. Zone I was defined as a circle with the radius that extends from the center of the optic disc to twice the distance from the center of the optic disc and the central macula. Posterior zone II was defined as a circle whose radius is three times the distance between the center of the optic disc and the center of the macula.

Persistent of ROP was defined as the lack of adequate regression of ROP. Recurrence was defined as arrest of anterior progression of retinal vasculature with new demarcation line, ridge, or extraretinal fibrovascular proliferation, with or without recurrence of plus disease [13].

### 2.3. Treatments and Follow-Ups

Infants were treated within 24 hours of diagnosis. The injection technique is described as follows. After the pupils were dilated with a combination of 0.5% tropicamide and 0.5% phenylephrine eye drops (Mydrin-P®, Santen Inc., Japan) the eyelids and conjunctiva were cleaned by 5% povidone iodine. A lid speculum was placed and an intravitreal injection with 0.25 mg/0.025 mL of ranibizumab was performed through pars plicata into the vitreous cavity with a 30-gauge needle inserted 1.0 mm posterior to the limbus of eyes under topical anesthesia with 0.5% proparacaine (Alcaine®, Alcon Laboratories Inc., USA). Vital signs were monitored throughout the entire procedure. The affected eye was given one drop of 0.3% ciprofloxacin 3 times a day for 5 days postoperatively. The patients were followed up at days 1, 2, 3, and 7 after IVR and then weekly until reaching 42 weeks postconceptual age (PCA).

In the cases exhibited with persistence/recurrence of ROP or peripheral retinal avascularity at PCA 42 weeks, treatment with laser photocoagulation was considered. All laser treatments were performed using an 810 nm diode laser (IRIS Medical Oculight SL 810 nm infrared laser; Iris Medical Inc., USA). Confluent laser burns, defined as laser burns less than half a burn width apart, were applied to the entire avascular retina. Repeated laser treatment to skip areas was carried out in one to two weeks after the primary laser treatment.

Then the treated patients were followed up at day 3, weekly or biweekly, or monthly to at least 24 weeks after retreatment. Extended follow-up was individually tailored according to response to treatment. Bilateral indirect ophthalmoscopy with scleral indentation was performed at each visit before and after treatment, and RetCam (Clarity Medical Systems, Pleasanton, CA, USA) wide-angle fundus imaging system was used to document fundus images of serial examinations.

## 3. Results

The demographic data of the patients are shown in Table 1. All these patients were transferred from outside hospitals. Among them, seventy-five percent (16/20) was male. The mean gestational age of these patients was 28.3 ± 1.6 weeks (range, 26–32 weeks) with the mean birth weight of 1221.3 ± 229.1 g (range, 900–1900 g). Four of the patients were from multiple birth pregnancies, and the remainder were singlets. All these patients had bronchopulmonary dysplasia, sepsis, and blood transfusions.

**Figure 1:**
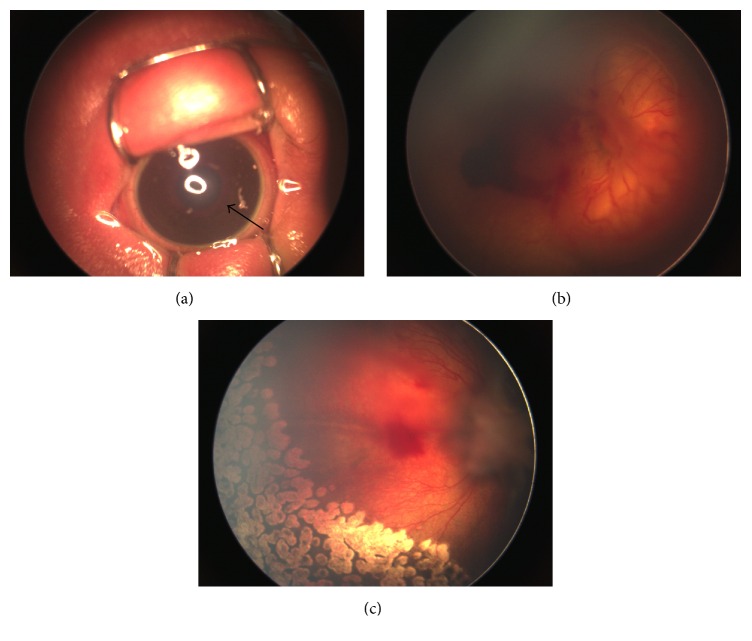
RetCam2 image of the right eye of patient number 17. (a) Anterior segment photography showing significant iris vascular engorgement (black arrow) and rigid pupil before IVR. (b) Before injection, fundus image showing prominent plus disease, vitreous hemorrhage, and fibrovascular proliferation at the posterior pole. (c) Fundus image 4 weeks after combination treatment of IVR and laser photocoagulation. An adequate regression of plus disease and significant absorption of vitreous hemorrhage was noted. A dense localized fibrous proliferation and mild ectopic macula was also noted.

On the baseline, all the eyes had poor pupil dilation, and 91.9% (34/37) eyes demonstrated iris vascular engorgement. The mean PCA of patients who received IVR was 34.6 ± 1.4 weeks (range, 32–38 weeks). Of the 37 eyes, 33 (89.2%) eyes had zone I and 4 (10.8%) eyes had posterior zone II disease. Two (5.4%) eyes demonstrated extraretinal fibrovascular proliferation before the initial treatment (Figure 1).

On day 7 after IVR, the rigid pupil and iris vascular engorgement of all these eyes disappeared. Thirty-one (83.8%) eyes demonstrated significant absorption of vitreous hemorrhage and four (10.8%) eyes showed partial absorption of vitreous hemorrhage, while two (5.4%) eyes did not show any change of the vitreous hemorrhage. Thereby, the two eyes that had no change in vitreous hemorrhage were defined as persistent of ROP and received laser therapy immediately. Adequate regression of dilation and tortuosity of posterior vessels was observed in sixteen (43.2%) eyes, and subtle regression was observed in the remainder.

On day 14 after IVR, no obvious change was observed in vitreous hemorrhage, compared with day 7. Twenty (54.1%) eyes demonstrated adequate regression of dilation and tortuosity of posterior vessels. Thereby, the remaining 15 eyes having subtle regression of dilation and tortuosity of posterior vessels were defined as persistent of ROP, and received laser therapy within 48 hours.

Among 20 eyes that had adequate regression of ROP, 6 eyes showed various extent of continued vascularization of the peripheral retina after IVR treatment. But none of them had vascularized Zone III. New demarcation line was exhibited in 16 (80%) of these 20 eyes during the follow-up. The mean recurrence time after IVR was 7.1 ± 1.6 weeks (range, 4–10 weeks). At PCA 42 weeks, four (10.8%) eyes demonstrated persistent peripheral retinal avascularity without any new demarcation line. According to the protocol, we conducted laser therapy for these eyes.

Thus, all eyes received first laser photocoagulation therapy after IVR. The mean time of received laser therapy after IVR was 4.8 ± 2.9 weeks (range, 1–10 weeks). Ten (27.0%) eyes received second laser therapy according to our protocol. The mean time of patients who received second laser therapy after IVR was 3.2 ± 0.8 weeks (range, 2–4 weeks). After the combination of IVR and laser photocoagulation treatment, all eyes demonstrated adequate regression of ROP.

All patients were followed up for a minimum of 28 weeks. The mean follow-up time was 39.3 ± 8.3 weeks (range, 28–52 weeks). At the end of follow-up, seven (18.9%) eyes exhibited fibrosis membrane, three (8.1%) eyes demonstrated mild ectopic macula, and the remainder had normal vascular pattern of the posterior fundus. All eyes were stable with attached retina without any further surgical intervention. No other significant ocular or systemic adverse effects related to IVR were observed in these patients during the follow-ups.

## 4. Discussion

Our study demonstrated that intravitreal injection of ranibizumab combined with laser photocoagulation might be effective for AP-ROP associated with vitreous hemorrhage. All the rigid pupils and iris vascular engorgement disappeared, and 94.6% eyes showed various degrees of absorption of vitreous hemorrhage after IVR treatment, which can improve the fundus visibility and might contribute to operability of following conventional laser photocoagulation therapy. After the combination treatment, all eyes demonstrated adequate regression of ROP and 92% eyes had a favorite anatomical result.

With the improvement of neonatal intensive care, more and more very preterm infants can survive, leading to the increasing incidence of AP-ROP [1]. However, the prognosis of AP-ROP is poorer than that reported for zone II ROP, despite frequent screening in high risk infants and timely confluent laser photocoagulation [4, 14, 15]. Unfavorable outcomes for zone I ROP range from 28.6% to 55% [3, 14, 15]. For those AP-ROP associated with vitreous hemorrhage eyes, the prognosis would be even poorer. As the poor fundus visibility, complete retinal ablation is usually impossible and the retinopathy may continue to progress. Previous reports described that vitreous hemorrhage is the major risk factor for development of unfavorable outcomes. Sanghi et al. reported that hemorrhages before laser treatment is one of the most significant risk factors for retinal detachment in AP-ROP despite confluent laser photocoagulation [4]. Kim et al. concluded in their study that the presence of pretreatment hemorrhage increased the odds of developing a retinal detachment (RD) by a factor of 10, and presence of vitreous organization increased the risk of RD by 16 times [6]. Therefore, the treatment options for these eyes are truly limited.

The purpose of laser photocoagulation is to reduce VEGF level produced by the avascular retina through ablating the periphery retina. Nowadays, anti-VEGF agents have been used as monotherapy or adjunctive therapy to laser photocoagulation, with effective results demonstrated [8–11, 16–19]. The majority of studies have reported the results of intravitreal injection of bevacizumab (IVB). There are a few studies that reported the results of IVR [10, 17, 18]. To the best of our knowledge, the present study is the first case series study about the treatment efficacy of combination of IVR and laser photocoagulation therapy in AP-ROP associated with vitreous hemorrhage patients.

In a recent retrospective research of 241 infants being followed up to over 65 weeks PCA, recurrence after IVB monotherapy for severe type 1 ROP was approximately 8.3% [20]. In another retrospective study, Yi et al. [18] treated 66 eyes of 33 premature infants diagnosed with type 1 ROP or AP-ROP with IVR as primary treatment. 87.9% eyes had total regression of ROP after a single injection. And 12.1% eyes had recurrence of ROP and received additional treatment. In our present study, only 54.1% eyes had adequate regression of ROP after the initial IVR treatment. The recurrence of ROP was observed in 43.2% (16/37) eyes, which is much higher than previous reports [8, 18, 20]. The reason of the lower rate of adequate regression and higher rate of recurrence of ROP after monotherapy of IVR in our study may probably be due to the fact that the patients we enrolled were more severe than other studies. But the recurrence time in our study ranged from 4 weeks to 10 weeks, which is quite similar to the other studies [18, 21]. Therefore, it seems that monotherapy of IVR is not sufficient in treating severe type ROP, such as AP-ROP associated with hemorrhage in particular. Close monitoring is important for early detection and timely retreatment of the recurrence of ROP and combination of laser photocoagulation therapy would be recommended.

An interesting finding is that, in our study group, 80% of patients were boys, indicating that boys may have predilection of severe ROP. However, we need to interpret this finding carefully. Our results might have been biased as the patients were all transferred from outside hospitals, and our sample size was small. They may not be able to represent the AP-ROP population. Although some previous studies reported that male gender is one of the predictors of treatment-requiring ROP [22, 23], we did not find any literature reporting the disparity in gender predilection to develop AP-ROP. Further prospective randomized studies may be needed to determine any gender predilection.

Safety is always of particular interest when considering the use of anti-VEGF agents in the treatment of infants, especially in our very vulnerable AP-ROP patients, as they are always associated with other systemic diseases and may still be in the process of organogenesis, in which VEGF still plays an essential role. Ranibizumab is an antibody fragment that has less molecular weight and better affinity to VEGF than bevacizumab [24]. This makes ranibizumab potentially more favorable in the treatment of infants with ROP with regard to efficacy and ocular and systemic safety profile. Recently, Wu et al. reported that serum VEGF levels in ROP patients were suppressed for two months after treatment with IVB, while VEGF levels were less affected after IVR treatment, which suggested that IVR could be a safer choice than IVB in the treatment of ROP [25]. In our present study, we did not observe any drug related systemic side effects during follow-up. But it remains important to be vigilant in the continued search for systemic complications and to conduct necessary clinical tests to identify any systemic complications.

On the other hand, the use of anti-VEGF agents for patients with ROP required attention to the risk of acute contraction of the proliferative membrane, thereby inducing or exacerbating RD. The development or progression of tractional RD is believed to be caused by a rapid neovascular involution with accelerated fibrosis and posterior hyaloid contraction, as a response to decreased levels of VEGF. There were a few case reports regarding progressive tractional RD after intravitreal injection of bevacizumab for ROP [26–28]. In our study, although seven eyes demonstrated fibrosis membrane, no patient had progressive fibrous traction after the injection.

Our study has several limitations worthy of consideration. The series is neither randomized nor prospective. The size of this cohort is relatively small, and all the data is from a single institution. Despite these limitations, the results suggest that combination of IVR and laser photocoagulation therapy can effectively treat AP-ROP with vitreous hemorrhage without additional vitreoretinal surgery and contributes to better anatomical results.

In conclusion, our study demonstrated that intravitreal injection of ranibizumab could be conducted as primary treatment of AP-ROP associated with vitreous hemorrhage, which can improve the fundus visibility, and followed by conventional laser photocoagulation therapy. Special attention must be paid to the risk of fibrous contraction and recurrence of ROP. Due to the limited case numbers, further randomized, prospective controlled trials are needed to determine the safety and definite efficacy and to improve our understanding of AP-ROP.

## Figures and Tables

**Table 1:** Characteristics of infants with AP-ROP associated with vitreous hemorrhage.

Patient number/eye		Gender	GA (weeks)	BW (g)	PCA (weeks), IVR	Zone before injection	Time after IVR (weeks), laser 1	Time after IVR (weeks), laser 2	Persistent ROP	Recurrence ROP
1	OD	M	27	1100	34	I	6			Y
	OS				34	I	8			Y
2	OD	M	28	1090	35	I	2	3	Y	
	OS				35	Posterior II	6			Y
3	OD	M	28	1400	33	Posterior II	7			Y
4	OD	M	28	1000	36	I	2	3	Y	
	OS				36	I	2		Y	
5	OD	F	27	1250	34	I	7			
	OS				34	I	7			Y
6	OD	M	28	900	36	I	2		Y	
	OS				36	I	2		Y	
7	OD	M	28	1250	36	I	2		Y	
	OS				36	I	2		Y	
8	OD	M	27	1125	33	I	9			
	OS				33	I	9			
9	OD	M	31	1500	36	I	2	3	Y	
10	OD	M	32	1500	38	I	2	4	Y	
	OS				38	I	2	4	Y	
11	OD	M	32	1900	36	I	2		Y	
	OS				36	I	2		Y	
12	OD	M	27	1130	34	I	8			Y
	OS				34	I	8			Y
13	OD	M	28	1010	34	I	6			Y
	OS				34	I	6			Y
14	OS	M	29	1200	35	I	1	2	Y	
15	OD	F	26	950	32	Posterior II	10			Y
	OS				32	Posterior II	10			Y
16	OD	M	28	1300	35	I	6			Y
	OS				35	I	2	4	Y	
17	OD	F	28	1100	34	I	1	2	Y	
	OS				34	I	4			Y
18	OD	M	27	1200	33	I	6			
	OS				33	I	6			Y
19	OD	F	28	1300	34	I	2	4	Y	
	OS				34	I	2	3	Y	
20	OD	M	29	1220	34	I	8			Y
	OS				34	I	8			Y

AP-ROP: aggressive posterior retinopathy of prematurity; BW: birth weight; F: female; GA: gestational age; M: male; OD: right eye; OS: left eye; PCA: postconceptual age; Y: yes.
